# Guanylate cyclase C limits systemic dissemination of a murine enteric pathogen

**DOI:** 10.1186/1471-230X-13-135

**Published:** 2013-09-02

**Authors:** Elizabeth A Mann, Eleana Harmel-Laws, Mitchell B Cohen, Kris A Steinbrecher

**Affiliations:** 1 Department of Pediatrics, University of Cincinnati College of Medicine, Cincinnati, OH, USA; 2 Division of Gastroenterology, Hepatology, and Nutrition, Cincinnati Children’s Hospital Medical Center, 3333 Burnet Ave, MLC 2010, 45229 Cincinnati, OH, USA

**Keywords:** Guanylate cyclase, Guanylyl cyclase, cGMP, Intestine, Colon, Attaching and effacing lesion, Epithelial cell apoptosis, Intestinal barrier function

## Abstract

**Background:**

Guanylate Cyclase C (GC-C) is an apically-oriented transmembrane receptor that is expressed on epithelial cells of the intestine. Activation of GC-C by the endogenous ligands guanylin or uroguanylin elevates intracellular cGMP and is implicated in intestinal ion secretion, cell proliferation, apoptosis, intestinal barrier function, as well as the susceptibility of the intestine to inflammation. Our aim was to determine if GC-C is required for host defense during infection by the murine enteric pathogen *Citrobacter rodentium* of the family *Enterobacteriacea*.

**Methods:**

GC-C^+/+^ control mice or those having GC-C genetically ablated (GC-C^−/−^) were administered *C. rodentium* by orogastric gavage and analyzed at multiple time points up to post-infection day 20. Commensal bacteria were characterized in uninfected GC-C^+/+^ and GC-C^−/−^ mice using 16S rRNA PCR analysis.

**Results:**

GC-C^−/−^ mice had an increase in *C. rodentium* bacterial load in stool relative to GC-C^+/+^. *C. rodentium* infection strongly decreased guanylin expression in GC-C^+/+^ mice and, to an even greater degree, in GC-C^−/−^ animals. Fluorescent tracer studies indicated that mice lacking GC-C, unlike GC-C^+/+^ animals, had a substantial loss of intestinal barrier function early in the course of infection. Epithelial cell apoptosis was significantly increased in GC-C^−/−^ mice following 10 days of infection and this was associated with increased frequency and numbers of *C. rodentium* translocation out of the intestine. Infection led to significant liver histopathology in GC-C^−/−^ mice as well as lymphocyte infiltration and elevated cytokine and chemokine expression. Relative to naïve GC-C^+/+^ mice, the commensal microflora load in uninfected GC-C^−/−^ mice was decreased and bacterial composition was imbalanced and included outgrowth of the *Enterobacteriacea* family.

**Conclusions:**

This work demonstrates the novel finding that GC-C signaling is an essential component of host defense during murine enteric infection by reducing bacterial load and preventing systemic dissemination of attaching/effacing-lesion forming bacterial pathogens such as *C. rodentium*.

## Background

*Citrobacter rodentium* (*C. rodentium*) are gram-negative bacteria of the family *Enterobacteriaceae*, and are natural enteric pathogens of mice. Related family members, enteropathogenic *Escherichia coli* (EPEC) and enterohemorrhagic *E. coli* (EHEC), are human pathogens that are a major cause of infectious diarrhea worldwide. Similar to these bacteria, *C. rodentium* are non-invasive. Instead, these attaching/effacing lesion-forming pathogens carry a set of virulence genes termed the locus of enterocyte effacement which enable close association with the apical membrane of intestinal cells, causing local destruction of microvilli and transfer of effector gene products via a type III secretion system [1]. In C57BL/6 mice and other strains that are considered resistant to *C. rodentium,* colonization results in a self-limiting infection which is cleared by both the innate and the adaptive immune systems. The course of infection is characterized by intestinal epithelial cell apoptosis, infiltration of inflammatory cells, mainly macrophage and neutrophils, and crypt hyperplasia, all of which are largely resolved within 3 weeks. In contrast, susceptible strains exhibit severe, often fatal, diarrhea which has been attributed at least in part to decreased expression of transporters important in chloride ion homeostasis [2] and to differences in the intestinal microbiota [3,4].

Guanylate cyclase 2C (encoded by *Gucy2c*, hereafter termed GC-C) is a transmembrane receptor present in the intestine which, in concert with its ligands guanylin (GN, encoded by *Guca2a*,) [5] and uroguanylin (UGN, encoded by *Guca2b*) [6], is known to regulate both chloride/bicarbonate ion secretion (via the cystic fibrosis transmembrane conductance regulator, CFTR) [7,8] and sodium absorption (via the Na^+^/H^+^ exchanger 3, NHE3, *Slc9a3*) [9]. The first step in this regulatory cascade is ligand activation of GC-C to generate the second messenger cyclic guanosine monophosphate (cGMP). Some strains of infectious enterotoxigenic *E. coli* produce the heat-stable enterotoxin, ST, which acts as a GC-C superagonist [10]. Unlike activation of GC-C by the endogenous ligands GN or UGN, robust overproduction of cGMP by ST-bound GC-C deregulates downstream signaling pathways and causes secretory diarrhea. Mice carrying a targeted disruption of *Gucy2c* have reduced levels of intestinal cGMP, and are resistant to ST [11,12]. Importantly, further studies of *Gucy2c* knockout (hereafter called GC-C^−/−^) mice have provided evidence for actions of GC-C beyond fluid and ion homeostasis in the gut. Signaling via GC-C has been implicated in intestinal epithelial cell cycle regulation, apoptosis, and progression to gastrointestinal cancer [13-15]. More recently, we have shown the presence of a defective intestinal epithelial barrier as well as sensitivity to DNA damage-induced cell death in mice lacking GC-C [16,17]. In a model of chemical-induced intestinal injury, GC-C^−/−^ mice exhibited a blunted intestinal inflammatory response that was accompanied by reduced cytokine expression [18]. Based on these findings, we utilized the *C. rodentium* model of enteric bacterial pathogen infection to investigate the hypothesis that GC-C effector pathways are an important aspect of host defense in the intestinal mucosa.

## Methods

### Mice

GC-C^−/−^ mice carrying a targeted deletion of *Gucy2c*, the gene encoding GC-C, have previously been described by us [11]. Heterozygous GC-C^+/−^ mice have been back-crossed to the C57BL/6 J strain (Jackson Laboratory, Bar Harbor, Me) for > 10 generations, and GC-C^−/−^ and GC-C^+/+^ mouse lines were generated by this process. As in our recent work [16,18], both GC-C^+/+^ and GC-C^−/−^ mouse lines were maintained in the same room of our animal facility under identical specific-pathogen free conditions. Mice of both sexes, aged 6–12 weeks were used, and experiments were performed on age- and gender- matched groups. All studies were approved by the Cincinnati Children’s Hospital Medical Center Institutional Animal Care and Use Committee.

### *C. rodentium* infection of mice

*C. rodentium* bacteria from frozen stocks of strain DBS 100 (gift of Philip M. Sherman, Hospital for Sick Children, Toronto, Canada) were grown on MacConkey’s agar (Becton, Dickinson and Company, Sparks, MD) overnight at 37°C. A single colony was then cultured in Luria-Bertani broth overnight at 37°C. We used optical density measurements at 600 nm to assess the concentration of bacteria, and confirmed colony forming units (CFU) by plating serial dilutions on MacConkey’s agar. Within each study, groups of GC-C^+/+^ and GC-C^−/−^ mice with free access to food and water were infected by oral gavage with the same freshly prepared mixture of C. rodentium (~1.5 × 10^9^ CFU *C. rodentium* in 100 μl sterile PBS per mouse). Stool from individual mice was collected and weighed every few days after gavage, beginning at day 4. To determine bacterial burden, stool was homogenized in sterile PBS (0.1 g stool/1 ml PBS) and serial dilutions were plated on MacConkey’s agar. Colonies were counted after overnight incubation at 37°C. The limit of detection was 5 × 10^2^ CFU/g stool. A mouse was excluded from further analysis if the level of *C. rodentium* was below the limit of detection at 4 days post-infection. PCR analysis of the *C. rodentium espB* gene was performed on representative colonies to confirm identity.

### Analysis of *C. rodentium* infected mice

Groups of mice were sacrificed at 4, 10, and 20 days after infection. Colon, feces, and liver were aseptically removed. A caudal piece (0.5 cm) of the distal colon was frozen in liquid nitrogen for subsequent RNA extraction, while the remaining section was split longitudinally into “swiss rolls” for formalin fixation/paraffin embedding and for frozen OCT embedding. Sections of liver were frozen in liquid nitrogen for RNA analysis or fixed in formalin/paraffin for histology. In addition, liver sections (trimmed to 0.1 g weight) were homogenized in 1 ml of sterile PBS, and plated on MacConkey’s agar. *C. rodentium* colonies were counted after incubation at 37°C overnight. The limit of detection for liver homogenates was 40 CFU/g and identity was confirmed by PCR analysis performed as above. Tissues from additional naïve (non-infected) age- and gender-matched mice were similarly obtained and processed.

### Histology, immunofluorescence and immunoblotting

H&E staining of intestinal and hepatic sections was done using standard techniques as previously described [18,19]. Images were obtained on an Olympus BX51 microscope equipped with an Olympus DP71 camera and DP Manager software. Measurements of crypt depth were taken on micrographs of all well-oriented crypts from H&E-stained “swiss roll” sections of distal colon using ImageJ version 1.38 (National Institutes of Health, Bethesda, MD). Stained slides (distal colon) were also examined to assess colitis severity in terms of disease scores composed of the degree of inflammation, hyperplasia, and infiltrate composition using a modified scoring system based on previous studies [18,20,21]. Briefly, this system assessed inflammation (scored 0–4 where 0 is no inflammation and 4 is severe ulceration and crypt abscess), hyperplasia (0 – 3 where 0 is no hyperplasia and 3 is severe hyperplasia with mucin depletion), and inflammatory composition (0 – 3 where 0 is none and 3 is substantial mononuclear cells with high neutrophil content). *C. rodentium* was localized in the distal colon by incubating OCT frozen sections with anti-*E. coli* Polyvalent 8 LPS antibody (1:500, Denka Seiken Co., Tokyo, Japan) as according to protocol and helpful advice from Dr. Bruce Vallance (University of British Colombia, Canada) [22]. Sections were then incubated with anti-swine CF-488A-conjugated IgG secondary antibody (1:2000, Sigma-Aldrich Corp., St. Louis, MO) and counter-stained with DAPI. Apoptosis was quantitated via immunofluorescence with cleaved caspase 3 antibody (CC3, antibody #9661, Cell Signaling Technology, Inc., Danvers, MA) as performed previously on OCT frozen sections [19,23]. Positive-stained intestinal epithelial cells were tabulated from 8–12 micrograph fields (original magnification 200x) per mouse. Immunofluorescence of E-cadherin (#13-1900, Life Technologies; Carlsbad, CA), claudin 2 (#51-6100, Life Technologies) and claudin 3 (#341700, Life Technologies) was performed on frozen sections in a similar manner. Tubulin, claudin 2, and claudin 3 immunoblotting was performed as previously described on extracts from homogenized colon.

### Real-time RT-PCR

Total RNA was isolated from frozen tissue using Tri Reagent (Molecular Research Center, Inc., Cincinnati, OH) according to the manufacturer's protocol. Integrity of the RNA was verified by visualization of 28S and 18S RNA after gel electrophoresis. Following cDNA preparation (starting quantity of 2.5 ug RNA) with random hexamers (Verso cDNA kit, Thermo Fisher Scientific, Inc., ABgene House, Epsom, UK), real-time PCR reactions using gene-specific primers were performed with Brilliant II SYBR Green QPCR mix (Stratagene, La Jolla, CA) in the Mx3000p thermocycler (Stratagene). Primer sequences for GC-C [24], NHE3 [25], and CXCL1 [19] and LBP [26] have been published previously. Additional primer sequences used in this report are given in Table 1. Values were normalized to expression of glyceraldehyde-3-phosphate dehydrogenase (GAPDH), and the average fold increase relative to the average level in gender-matched naïve mice was calculated using the comparative threshold cycle method (∆∆Ct).

**Table 1:** Real time RT-PCR primers

**Primers**	**Sequences**
Gn	For 5’- CAC TGT GCA GGA TGG AGA C-3’
	Rev 5’-CTC GGC GTT GGG TTT CT-3’
CFTR	For 5’-GCA CAG CAG CTC AAA CAA CTG GAA-3’
	Rev 5’-TTC TCA TTT GGA ACC AGC GCA AGC-3’
IL-22	For 5’-AAA CTG TTC CGA GGA GTC AGT GCT
	Rev 5’-GCT GAG CTG ATT GCT GAG TTT GGT-3’
CXCL9	For 5’-TTT CAT CAC GCC CTT GAG CCT AGT-3’
	Rev 5’-TTT GGT GAC GTG AGC CTC AGA AGT-3’
GAPDH	For 5’-ACC ACA GTC CAT GCC ATC AC-3’
	Rev 5’-TCC ACC ACC CTG TTG CTG TA-3’

### Measurement of intestinal permeability

Intestinal permeability was assessed by measuring movement of a fluorescent tracer (4 kDa FITC-dextran, Sigma-Aldrich, St. Louis, MO) from the gut lumen to the blood. Uninfected and day 4 *C. rodentium* infected mice of both GC-C^+/+^ and GC-C^−/−^ genotypes were orally gavaged with 200ul of 70 mg/ml FITC-dextran dissolved in water. Mice were given FITC-dextran solution four hours prior to blood collection at sacrifice. Serum samples were diluted and read in a 96 well plate at 485 excitation/535 emission along with a FITC-dextran standard curve. The calculated serum concentration of FITC-dextran was interpreted as an indication of intestinal permeability.

### Bacterial DNA and quantitative PCR analysis

Bacterial DNA was isolated with the QIAamp DNA Stool Mini Kit (QIAGEN, Valencia, CA) according to manufacturer’s instructions for pathogen DNA, including the optional 95°C incubation step. Stool from individual adult mice was weighed, frozen at −80°C, and homogenized directly in ASL lysis buffer (QIAGEN) prior to DNA extraction. Genomic DNA from cultures of the laboratory *Escherichia coli* strain, DH5α, was also prepared using this kit. Integrity of DNA was checked by agarose gel electrophoresis, and the concentration determined using the Quant-iT dsDNA Broad-Range Assay Kit (Invitrogen, Molecular Probes, Inc., Eugene, OR). To quantify bacteria, real-time PCR was performed using Brilliant II SYBR Green QPCR mix (Stratagene) and published sets of group- or family-specific 16S ribosomal RNA (rRNA) gene primers as follows: *Eubacterium rectales*/*Clostridiae cocoides*[27], *Enterobacteriacea*[27], *Atopobium*[28]*Lactobacillus*[28], *Bacteroides-Prevotella-Porphyromonas*[28] and *Bifidobacteria*[28]. For determining total bacterial load, universal 16S rRNA gene primers that recognize all eubacteria were used [27]. A standard curve of serial dilutions of a known amount of DH5α DNA was generated, and used to interpolate the quantity of bacteria from each experimental DNA sample. To assess the relative quantity of each group the ∆Ct method was used with normalization by total bacteria values [29].

### Data analysis

Categorical variables were evaluated by Fisher exact test. Differences in continuous variables were compared using the Mann–Whitney test or 2-tailed Student *t* test. For all analyses, statistical significance was set at *P* ≤ 0.05 and was determined using Prism Version 5.03 (GraphPad Software, Inc., San Diego, CA).

## Results

### Mice lacking GC-C exhibit increased load of *C. rodentium* but retain the ability to clear the infection

**Figure 1:**
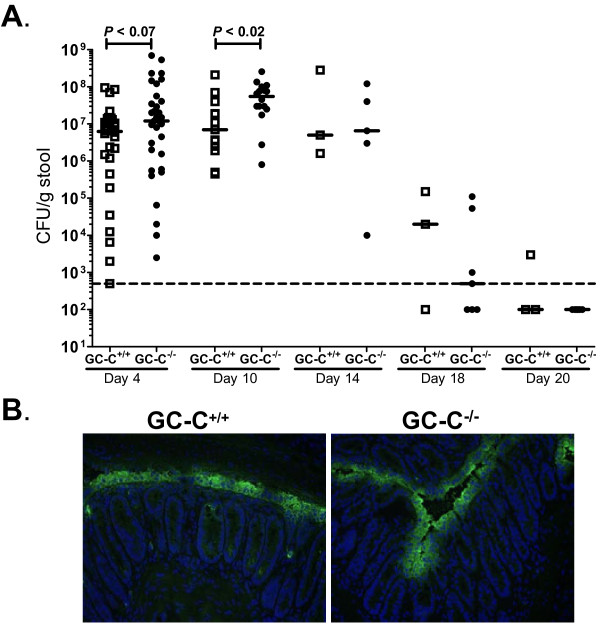
**GC-C**^**−/− **^**mice have an increased load of *C. rodentium *compared to GC-C**^**+/+ **^**but are still able to clear the infection. A**: Increasing numbers of *C. rodentium* were detected in stool from GC-C^−/−^ mice such that by day 10 there were significantly greater numbers of *C. rodentium* present relative to GC-C^+/+^. Note that both genotypes were able to clear the infection by day 20. Each time point consisted of groups of 3–26 mice. Solid line represents median value. Dotted line indicates limit of detection (500 CFU/g stool). Samples below limit of detection were arbitrarily given a value of 100. **B**: Representative photomicrographs of sections of distal colon of infected GC-C^−/−^ and GC-C^+/+^ mice labeled with LPS specific antibody (green) exhibit similar bacterial localization at day 10 p.i. Images were counterstained with DAPI (blue). Original magnification was 400X.

Mice were infected with *C. rodentium* (1.5 × 10^9^ CFU) by oral gavage. Colonization was monitored by stool culture beginning at 4 days post-infection (p.i.). The results from several experiments are summarized in Figure 1A. For both genotypes the number of *C. rodentium* CFU recovered from stool varied widely at 4 days p.i., rose to a plateau between days 10 and 14, and then declined to the lower limit of detection by day 20. This is similar to the time course for infection in C57BL/6 mice previously reported in the literature [30,31]. There was a strong trend toward increased *C. rodentium* load in GC-C^−/−^ mice by 4 days p.i. but this narrowly missed statistical significance (*P* = 0.07). At 10 days p.i., the median number of *C. rodentium* present in the stool of GC-C^−/−^ mice was 8 times higher than GC-C^+/+^ (GC-C^+/+^, 7.0 × 10^6^ vs. GC-C^−/−^, 5.5 × 10^7^ CFU/g stool, *P* ≤ 0.02). This was not the case during the latter stages of the infection course when adaptive immunity typically reduces *C. rodentium* numbers and eventually clears the pathogen. We used immunofluoresent staining with antibodies reactive to *C. rodentium* lipopolysaccharide (LPS) to localize the bacteria in distal colon tissue sections at days 4 (data not shown) and 10 p.i. (Figure 1B). At both time points, there was no difference in localization of the pathogen as there was apparent staining at the epithelial cell surface of both genotypes. There were also occasional crypts that exhibited staining along the upper crypt epithelium in both genotypes.

**Figure 2:**
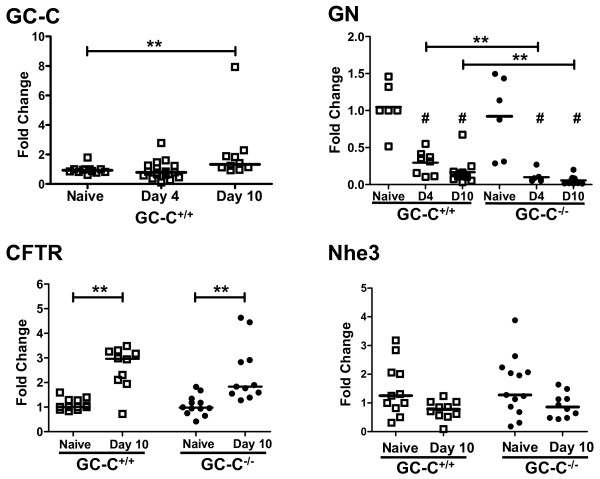
**Differential expression of GC-C, GN, and ion transport genes in the distal colon of naïve and infected mice.** While GC-C expression was slightly increased at 10 p.i., GN was substantially decreased in GC-C^+/+^ mice and to an even greater degree in GC-C^−/−^ animals at both day 4 and day 10 p.i. In both genotypes, CFTR expression was elevated while NHE3 was unchanged. Fold change values are relative to naive GC-C^+/+^ gut. Solid line represents median value. (**P ≤ 0.007; #*P* ≤ 0.02 naïve vs. infected of the same genotype; n = 6-13 mice per group; D4, day 4 p.i.; D10, day 10 p.i.)

Mice of the C57BL/6 J genetic background do not respond to *C. rodentium* infection with grossly obvious clinical symptoms such as weight loss or diarrhea [2,32]. As expected, we found this to be the case in GC-C^+/+^ mice and, in fact, there were no symptoms of diarrhea or loss of body weight in GC-C^−/−^ mice as well (data not shown). Real-time RT- PCR analysis showed that the level of GC-C expression in the distal colon of GC-C^+/+^ mice was modestly elevated at 10 p.i. (Figure 2). Production of GN, the most abundant colonic ligand of GC-C, was substantially decreased beginning at day 4 of infection in both genotypes, although this was significantly greater in infected GC-C^−/−^ mice relative to infected wildtype animals. It was notable that loss of Gn expression begins at infection day 4, prior to the pronounced epithelial hyperplasia or goblet cell loss typically seen by us and others in *C. rodentium* infected mice at later disease time points. GC-C generates cGMP that in turn controls the activity of several epithelial ion transporters, many of which are transcriptionally regulated during *C. rodentium* infection [32,33]. CFTR chloride channel mRNA increased at day 10 p.i. in mice of both genotypes compared to uninfected mice while there was no difference in expression of the Na^+^/H^+^ exchanger NHE3 (Figure 2).

### Epithelial hyperplasia during *C. rodentium* infection is not affected by deletion of GC-C^−/−^

**Figure 3:**
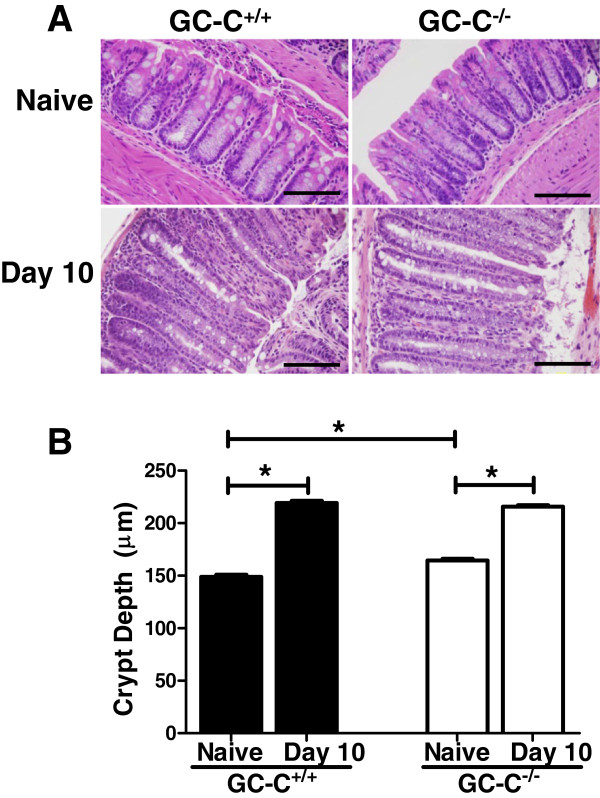
**Colonocyte hyperplasia is increased in response to infection to a similar extent in both GC-C**^**+/+ **^**and GC-C**^**−/− **^**mice. A**: Analysis of distal colon of naïve and day 10 p.i. mice revealed that there was no substantial difference in histopathology or epithelial cell hyperplasia in GC-C^+/+^ and GC-C^−/−^ mice. Scale bars = 100 μm, original magnification 400X. **B**: Quantitative analysis revealed a significant difference in crypt depth in the distal colon of uninfected GC-C^−/−^ mice compared to GC-C^+/+^ mice (n = 4 per group with a minimum of 30 crypts counted per mouse). In response to infection, the distal colons of both GC-C^+/+^ mice and GC-C^−/−^ mice show a significant increase in crypt depth (n = 12 per group with a minimum of 16 crypts per mouse). Data is presented as the mean with SE. (*P ≤ 0.0001, Student *t* test).

Bacterial pathogen infection of the colon is often associated with a derangement in epithelial homeostasis as reflected by altered crypt architecture and cell death. For example, crypt hyperplasia is a hallmark of the host response to cell death and inflammation caused by *C. rodentium* infection [34]. Histological examination of *C. rodentium* infected mice at day 10 p.i. revealed significant epithelial hyperplasia in both GC-C^+/+^ and GC-C^−/−^ mice (Figure 3A). Marked inflammation and immune cell infiltrate were also apparent in both genotypes and was not affected by the absence of GC-C (histologic disease score for GC-C^+/+^, 5.2 ± 0.8 vs. GC-C^−/−^, 6.0 ± 0.7, mean with SE, n = 12 mice per group, *P* = 0.49, Student t test). Depth measurements were taken in well-oriented crypt sections from the distal colon of naive and day 10 infected mice (Figure 3B). A modest but significant increase in crypt depth was found in naive GC-C^−/−^ distal colon compared to wild type (164 ± 1.67 μm vs. 146 ± 1.92 μm, mean ± SEM, *P* ≤ 0.0001). This is in agreement with earlier studies by us and others showing increased crypt depth in the colon of mice lacking GC-C or GC-C-activating ligands such as GN [35,13]. *C. rodentium* infection significantly increased crypt depth in both genotypes as measured at day 10 p.i. However, no significant difference in colonic crypt depth between infected GC-C^+/+^ (219 ± 2.09 μm) and infected GC-C^−/−^ (216 ± 1.61 μm) mice was detected.

### GC-C regulates colonic epithelial apoptosis during bacterial pathogen infection

**Figure 4:**
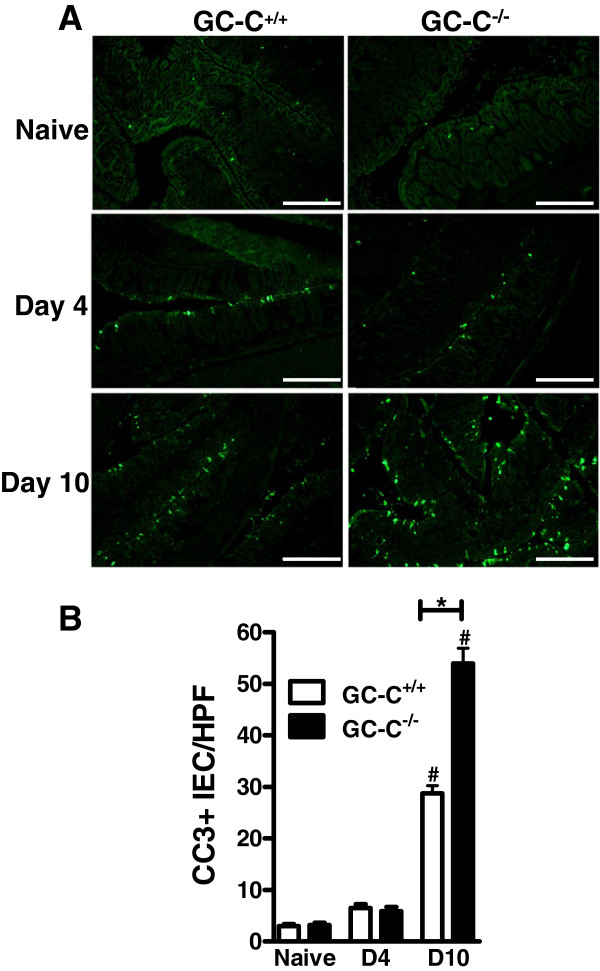
***C. rodentium *infection elicits colonocyte apoptosis to a greater degree in GC-C**^**−/− **^**mice than in GC-C**^**+/+**^**. A**: Immunofluorescent detection of the apoptotic marker cleaved caspase 3 (CC3) in representative sections of distal colon. Elevated numbers of positively stained epithelial cells are visible in sections from mice at days 4 and 10 following infection compared to naïve mice, with the highest staining seen in GC-C^−/−^ gut at day 10 p.i. Scale bars = 200 μm. **B**: A significant increase in the mean number of CC3-positive intestinal epithelial cells (CC3+ IEC) per high power field (HPF) was found in day 10 p.i. distal colon of GC-C^−/−^ compared to GC-C^+/+^ mice. Data is presented as the mean with SE. (*P ≤ 0.0001; Student *t* test; #P ≤ 0.0001 naïve vs. day 10 p.i.; naïve and day 4 mice n = 4 per group; day 10 mice n = 12 per group; D4, day 4 p.i.; D10, day 10 p.i.).

We have previously shown that GC-C activity plays an important role in regulating epithelial apoptosis during radiation and chemical challenge [17,18]. *C. rodentium* infection is associated with increased epithelial cell death in the intestine [1]. Therefore, we quantitated staining of epithelial cleaved caspase 3 as a marker of apoptosis in colonic sections from naïve, day 4, and day 10 infected mice. As expected, there was a similar, low level of cell death in naïve GC-C^+/+^ and GC-C^−/−^ colon (Figure 4A). While there was only a minimal increase in *C. rodentium* infected mice of either genotype at day 4 p.i., cleaved caspase 3 staining was highly elevated at day 10 p.i. in GC-C^−/−^ mice as compared to wildtype controls. Notably, widespread and pervasive epithelial cell death in GC-C^−/−^ colon was apparent. Quantitation of cleaved caspase 3-positive epithelial cells demonstrated nearly a doubling of apoptosis in GC-C^−/−^ mice relative to GC-C^+/+^ animals at day 10 p.i. (Figure 4B). These data clearly indicate that GC-C is essential for epithelial cell resistance to cell death induced by attaching and effacing lesion forming bacteria.

### Citrobacter infection induces barrier dysfunction in the absence of GC-C

**Figure 5:**
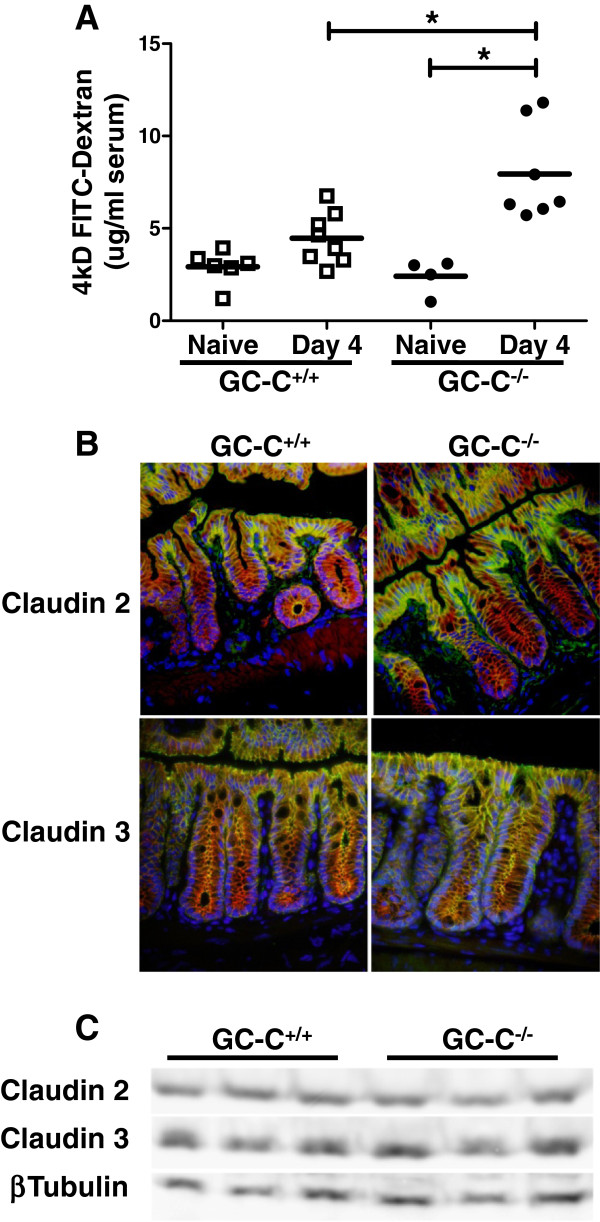
**GC-C is essential to maintain intestinal barrier function during *C. rodentium *infection. A**: GC-C^+/+^ and GC-C^−/−^ mice were given a FITC-dextran tracer by oral gavage on day 4 p.i. and serum fluorescence was measured four hours later. Relative to naïve mice as well as infected GC-C^+/+^ animals, GC-C^−/−^ mice have a substantial and significant increase in tracer movement from the gut lumen into the blood indicating a loss of intestinal barrier function. (*P ≤ 0.05; n = 4 – 8 mice per group). **B**: Immunofluorescent staining of claudin 2 or claudin 3 (green) with E-cadherin (red) and DAPI (blue) counter stains reveals no apparent differences between infected control and GC-C^−/−^ mice at day 4 p.i. **C**: Immunoblotting of claudin 2 and claudin 3 shows similar protein levels in extracts from distal colon of mice infected by *C. rodentium* for 4 days. βTubulin is shown to demonstrate equal loading.

In the context of *C. rodentium* infection, increased hyperplasia and cell death at the height of infection (~day 10 p.i.), such as that measured in GC-C^+/+^ and GC-C^−/−^ mice, can enhance epithelial permeability. Previous work by us and others indicates that GC-C may be important in regulating intestinal barrier function as its deletion correlates with loss of epithelial tight junction stability in the bowel [16,36]. Therefore, we hypothesized that subtle defects in colonic barrier function may be apparent in GC-C^−/−^ mice early in the course of *C. rodentium* infection, prior to the widespread epithelial damage present by day 10 p.i. In order to track potential movement of luminal contents through the epithelial cell layer and into the serosal compartment, we orally gavaged uninfected and day 4 p.i. GC-C^+/+^ and GC-C^−/−^ mice with a 4 kDa fluorescent tracer (FITC-dextran) four hours prior to necropsy. Unlike GC-C^+/+^ mice, animals lacking GC-C had a substantial loss of barrier function following 4 days of *C. rodentium* infection as indicated by increased serum FITC fluorescence relative to infected GC-C^+/+^ mice (Figure 5A). Claudin proteins are essential for tight junction stability and loss of specific claudin isoforms is associated with barrier dysfunction during *C. rodentium* infection [16,36-38]. Therefore, we selected two relevant claudin proteins, claudins 2 and 3, and determined their localization and expression levels in *C. rodentium*-infected wildtype and GC-C^−/−^ mice at day 4 p.i. Colonic sections (Figure 5B), counter-stained with E-cadherin and DAPI, showed no genotype-dependent differences in cell membrane localization of either claudin 2 or claudin 3. Furthermore, immunoblotting demonstrated that each was present in similar quantities within the colon of GC-C^+/+^ and GC-C^−/−^ mice (Figure 5C). Collectively, these data demonstrate that GC-C is essential for preserving colonic barrier function beginning at the earliest stages of enteric bacterial infection but that additional studies will be necessary to define the molecular mechanisms that drive this loss of function in GC-C^−/−^ mice.

### GC-C limits systemic dissemination of *C. rodentium*

**Figure 6:**
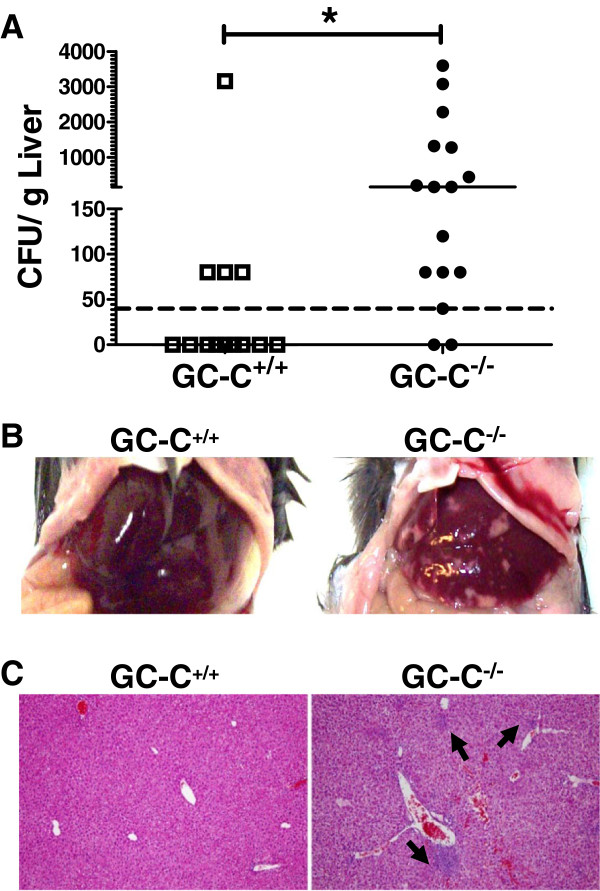
**Dissemination of *C. rodentium *from the intestine causes liver injury in GC-C**^**−/− **^**mice at day 10 following infection. A**: In colonized mice, GC-C^−/−^ livers had significantly more *C. rodentium* CFU/g as compared to GC-C^+/+^ livers. (*P ≤ 0.003). **B**: Gross areas of necrosis were apparent in the livers of some GC-C^−/−^ mice at necropsy while none were seen in GC-C^+/+^ livers. **C**: Representative photomicrographs illustrate the presence of lymphocyte aggregates (black arrows) and areas of hemorrhage visible only in GC-C^−/−^ mice (original magnification 200x).

A low level of bacterial translocation from the intestine to the blood, liver, and other organs occurs during the course of experimental *C. rodentium* infection [39,40]. The barrier dysfunction and increased apoptosis found in GC-C^−/−^ mice during infection suggested that these mice may fail to sequester the pathogen in the intestine. We therefore assessed *C. rodentium* translocation out of the intestine by plating liver homogenates from colonized mice on MacConkey’s agar. At 4 days p.i., no live bacteria were detected in homogenized liver from either genotype. However, by day 10 p.i. there were significantly more GC-C^−/−^ mice that exhibited colony growth: 31% (4/13) of GC-C^+/+^ livers were positive for *C. rodentium* compared to 88% (14/16) of GC-C^−/−^ livers (*P* ≤ 0.003, Fisher’s exact test). The number of colony forming units of *C. rodentium* recovered from day 10 p.i. liver samples was also significantly increased in mice lacking GC-C (Figure 6A). By day 20, colonies were no longer detectable from livers of GC-C^−/−^ mice, consistent with the time course of bacterial clearance from the stool.

We next performed a histological survey of liver tissue from wildtype and GC-C^−/−^ mice. Day 4 p.i. liver histology revealed mild inflammatory infiltrate with no indication of increased severity in GC-C^−/−^ mice as compared to GC-C^+/+^ animals (data not shown). Consistent with a lack of intestinal containment of *C. rodentium*, focal areas of necrosis at day 10 p.i., were noted in the liver of GC-C^−/−^ mice upon necropsy whereas none could be seen in the livers of any mice of the GC-C^+/+^ group (Figure 6B). This damage was apparent histologically as well, and included multiple areas of inflammatory infiltrate visible upon microscopic examination of GC-C^−/−^ liver sections (Figure 6C).

**Figure 7:**
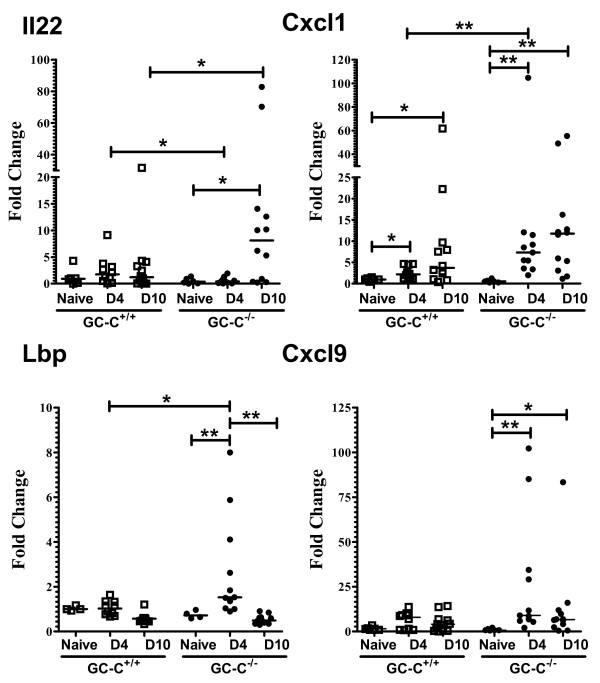
**Pro-inflammatory gene expression is enhanced in GC-C**^**−/− **^**livers at 4 and 10 days post-infection.** Real time RT-PCR was used to assess differences in liver gene expression from naive and infected mice at days 4 and 10 p.i. At day 4, levels of CXCL1, CXCL9, and LBP were increased in liver from GC-C^−/−^ mice. Consistent with the obvious liver damage seen in the GC-C^−/−^ mice at day 10, levels of IL-22, CXCL1, and CXCL9 were significantly elevated. All values are relative to the level in uninfected GC-C^+/+^ liver. Line represents median value. (**P ≤ 0.005, *P ≤ 0.05, n = 4-10 mice per group; D4, day 4 p.i.; D10, day 10 p.i.).

Our analysis then focused on gene expression in livers of mice infected with *C. rodentium* with the expectation that inflammation in GC-C^−/−^ mice would coincide with elevated activation of pro-inflammatory genes. Relative to naïve mice, we found that there was increased liver expression of inflammatory cytokines and chemo-attractants such as IL-22, CXCL1, and CXCL9 and that this increase was significantly more in mice lacking GC-C (Figure 7). Similarly, lipopolysaccharide binding protein (LBP) was elevated at day 4 p.i. in GC-C^−/−^ liver. These data are consistent with initial barrier dysfunction occurring at day 4 p.i. and suggests that intestinal barrier loss and circulation of luminal contents precedes translocation of live bacteria from the gut lumen in these mice.

### Commensal microflora composition is altered in gut of naïve mice lacking GC-C

**Figure 8:**
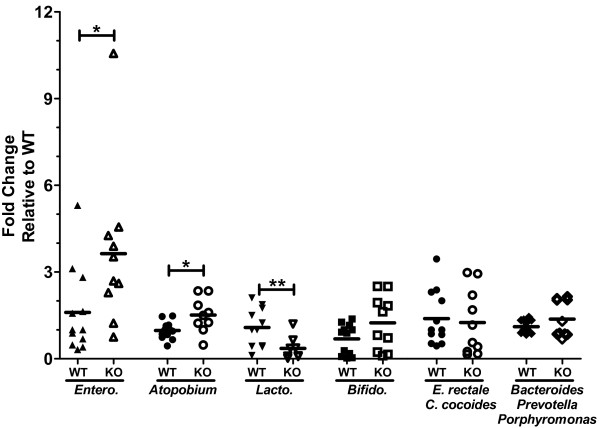
**The composition of commensal bacteria is altered in naive GC-C**^**−/− **^**mice relative to GC-C**^**+/+**^**.** Real time PCR of bacterial genomic DNA extracted from stool was performed using family-specific bacterial 16S rRNA gene primers. There was significant enrichment of *Enterobacteriaceae* and *Atopobium* in GC-C^−/−^ compared to GC-C^+/+^ while the level of *Lactobacillus* was decreased. Line represents median value. (*Entero*., *Enterobacteriaceae; Lacto., Lactobacillus; Bifido., Bifidobacteria; E. rectale, Eubacterium rectale; C. cocoides, Clostridiae cocoides;* WT, wildtype GC-C^+/+^; KO, GC-C^−/−^; *P < 0.03, ** *P* < 0.005, n = 10–12 per group).

Commensal microflora load and diversity are strongly influenced by epithelial ion transport in the intestine [41-44]. Importantly, the pathogenicity of *C. rodentium* is affected by the complement of commensal bacteria in the gut [3,4]. We therefore used bacterial DNA extracted from stool to monitor any potential differences in bacterial load or composition in naive GC-C^−/−^ mice compared to GC-C^+/+^. Bacterial load was quantified by PCR with universal primers that recognize eubacterial 16S ribosomal RNA genes, and then normalized to stool weight. We initially found a statistically significant decrease in stool bacterial load in GC-C^−/−^ mice (GC-C^+/+^ , 2.3 × 10^7^ copies/gram stool vs. GC-C^−/−^, 1.0 × 10^7^, n = 10–12, *P* ≤ 0.002, Mann Whitney test). We next utilized group and family-specific 16S gene primers to assess microflora diversity from GC-C^+/+^ stool compared to GC-C^−/−^ stool (Figure 8). In stool from GC-C^−/−^ mice there was increased levels of *Enterobacteriacae* (1.0 vs. 3.1 fold-change, GC-C^+/+^ vs. GC-C^−/−^, *P* ≤ 0.03) and *Atopobium* (1.0 vs. 1.5, *P* ≤ 0.03) as well as decreased presence of the *Lactobacillus* group (1.0 vs. 0.23 fold-change, *P* ≤ 0.01) compared to GC-C^+/+^ mice. No differences were found with primers recognizing *Bifidobacteria*, *Eubacterium rectale*/*Clostridiae cocoides,* and the *Bacteroides-Prevotella-Porphyromonas* group.

## Discussion

Targeting of GC-C by ST results in severe secretory diarrhea and is a significant cause of morbidity and mortality in children of developing countries. It has long been speculated that this GC-C-conferred susceptibility to some forms of enteric infection is offset by a critical role for this receptor in intestinal homeostasis. We and others have suggested that GC-C is important for epithelial ion transport, cell proliferation, and barrier function and that GC-C modulates intestinal disorders ranging from cystic fibrosis to gastrointestinal cancer to intestinal injury and inflammation via these effects [14,16-18,45]. Here, we extend the role of GC-C in the intestine by demonstrating that, although it is exploited by ST-producing ETEC, this pathway is highly protective during enteric infection by attaching/effacing bacterial pathogens which do not produce ST toxin. Specifically, we show that GC-C is required to minimize bacterial load during the early to middle phases of *C. rodentium* infection. We also demonstrate that GC-C activity is essential for maintaining the integrity of the intestinal epithelial barrier, both by reducing permeability early in infection as well as by suppressing epithelial apoptosis at later time points. The important role of GC-C in controlling pathogen load and intestinal barrier function is likely instrumental in minimizing systemic dissemination of attaching/effacing lesion forming bacteria such as *C. rodentium*. Furthermore, we demonstrate several additional novel aspects of epithelial GC-C/cGMP signaling. First, in the context of the C57BL/6 genetic background, infection by attaching/effacing bacteria such as *C. rodentium* does not significantly disrupt intestinal fluid homeostasis in the absence of GC-C. Second, loss of GC-C has no apparent impact on the hyperplasia of epithelial crypts that occurs in response to infection by attaching/effacing bacterial pathogens. Third, Gn expression is profoundly reduced during *C. rodentium* infection and this occurs prior to epithelial hypertrophy or substantial immune cell infiltration. Fourth, GC-C regulates the number and composition of intestinal commensal microflora in naïve mice.

Several multifunctional effector proteins (such as EspF and Map) common to *C. rodentium* and other attaching/effacing pathogens, cause host epithelial cell apoptosis as well as the disruption of intercellular tight junctions [37,46,47]. Bacterial translocation is associated with the intestinal cell death, inflammation, and barrier dysfunction seen during experimental *C. rodentium* infection [31,39,48]. We have previously reported increased radiation-induced intestinal epithelial cell apoptosis and epithelial barrier dysfunction in response to LPS challenge that resulted in translocation of commensal bacteria in GC-C^−/−^ mice [16,17]. In the current study, we show both a loss of barrier integrity in the intestine at early stages of infection and a significant elevation in intestinal epithelial apoptosis in GC-C^−/−^ mice that culminates in translocation of *C. rodentium* out of the intestine. While this correlates with the increased bacterial load in stool from GC-C^−/−^ at day 10, it is notable that we did not find major differences in the localization of *C. rodentium* colonization within the colon of GC-C^−/−^ mice as compared to GC-C^+/+^. At day 4, prior to translocation of live bacteria, there was significantly increased intestinal permeability in GC-C^−/−^ mice and this correlated with elevated expression of chemokine and acute phase genes in the liver as compared to GC-C^+/+^. Collectively, this work indicates that GC-C activity is an essential component of epithelial barrier function and anti-apoptosis and is required for multiple aspects of intestinal barrier function during enteric infection.

There is increasing evidence that complex, reciprocal crosstalk between the host and its commensal microflora has a broad and profound impact on gastrointestinal mucosal immunity and is a critical factor in defining susceptibility to cancer, inflammation, obesity as well as infection by bacterial pathogens [49]. Fecal transplant from a *C. rodentium*-resistant mouse strain reduced mortality in a susceptible murine genetic background [3,4]. In addition, treatment with the probiotic bacteria *Lactobaccillus* promoted survival of *C. rodentium*-infected neonatal mice [50], while exposure to a stressor (physical restraint) both altered gut microbial composition and increased colonization by *C. rodentium*[51]. Many factors shape the influence of commensal microflora on colonization resistance to a pathogen, and may include direct inhibition by bacteriocins (antimicrobials generated by bacteria), bacterial metabolites, or competition for nutrients [49]. Alternatively, the abundance of closely related family members may be indicative of the potential success of a pathogen from the same family with related growth characteristics [52]. Because an imbalance in intestinal microflora often occurs in mice with deregulated ion transport pathways [41,43], we investigated whether this may contribute to the increased *C. rodentium* colonization noted in GC-C^−/−^ mice. Here, we have demonstrated that loss of GC-C has a significant impact on the abundance and systemic dissemination of an enteric bacterial pathogen and we further show that naïve GC-C^−/−^ mice have a significant imbalance in intestinal bacterial species relative to their GC-C^+/+^ counterparts. In addition to a decrease in potentially protective *Lactobacillus*, the *Enterobacteriaceae* family colonizes the gut of naïve GC-C^−/−^ mice at higher levels than GC-C^+/+^, as does the *Enterobacteriaceae* family member *C. rodentium* during the early stages of the infection course. It is possible that GC-C activity regulates outgrowth of commensal bacteria as well as *C. rodentium* by affecting epithelial mucus layer dynamics, cell surface or luminal pH, or the ion/solute-dependent aspects of bacterial metabolism and growth. Additional studies will be necessary to define the mechanism through which GC-C influences the composition and load of commensal bacteria.

External factors, such as variation in diet and animal husbandry conditions at any given research institute, impact the composition of gut microflora and can have a dominant influence on disease model phenotype [53-55]. Therefore, genetically-modified mice used as models of gastrointestinal pathology such as bacterial infection, inflammation, obesity, and tumor susceptibility reflect the interaction between investigator-imposed genetic modifications and institution- or animal colony-dependent microflora. Further characterization and study of the microbiome of the GC-C^−/−^ intestine will be necessary to establish its specific role in susceptibility to bacterial infection as well as its putative broader impact on gastrointestinal pathophysiology.

## Conclusions

In summary, we have identified a protective role for GC-C signaling in defense against an attaching/effacing lesion-forming bacterial pathogen. Our studies have shown that GC-C is required to maintain an effective epithelial barrier and to suppress systemic dissemination of *C. rodentium*. We also demonstrate an imbalance in commensal microflora in naïve GC-C^−/−^ mice relative to GC-C^+/+^ animals from the same colony and speculate that this is an important component in the increased colonization of enteric pathogens in mice lacking GC-C.

## Abbreviations

GC-C: Guanylate cyclase 2C, GUCY2C; GN: Guanylin; CFTR: Cystic fibrosis transmembrane conductance regulator; NHE3: Na^+^/H^+^ exchanger 3; Cxcl1: Chemokine (C-X-C motif) ligand 1; Cxcl9: Chemokine (C-X-C motif) ligand 9; LBP: Lipopolysaccharide binding protein; IL-22: Interleukin 22.

## Competing interests

The authors affirm that they are not entered in any relationships with any organization or entity having a direct financial or personal interest in the subject matter or materials discussed in the article.

## Authors’ contributions

EAM, EHL, and KAS participated in study design, performed experiments and interpreted data. EAM and KAS wrote and edited the paper. MBC contributed essential reagents, participated in study design, and edited the paper. All authors read and approved the final manuscript.

## Pre-publication history

The pre-publication history for this paper can be accessed here:

http://www.biomedcentral.com/1471-230X/13/135/prepub
